# Evaluation of afoxolaner chewables to control flea populations in naturally infested dogs in private residences in Tampa FL, USA

**DOI:** 10.1186/s13071-015-0897-z

**Published:** 2015-05-24

**Authors:** Michael W. Dryden, Vicki Smith, Monica Chwala, Emery Jones, Lisa Crevoiserat, Jennifer C. McGrady, Kaitlin M. Foley, Paula R. Patton, Anthony Hawkins, Doug Carithers

**Affiliations:** Department of Diagnostic Medicine/Pathobiology, Kansas State University, Manhattan, KS 66506 USA; Merial, Inc., 3239 Satellite Blvd., Duluth, GA 30096 USA

**Keywords:** *Ctenocephalides felis felis*, Cat flea, Dogs, Field study, Tampa FL, Afoxolaner, Flea, Flea control

## Abstract

**Background:**

A study was conducted to evaluate the effectiveness of afoxolaner chewables to control flea populations in naturally infested dogs in private residences in Tampa FL, USA. Evaluations of on-animal and premises flea burdens, flea sex structure and fed-unfed premises flea populations were conducted to more accurately assess flea population dynamics in households.

**Methods:**

Thirty seven naturally flea infested dogs in 23 homes in Tampa, FL were enrolled in the study and treated with afoxolaner chewables. Chewables (NexGard® Chewables; Merial) were administered according to label directions by study investigators on study day 0 and once again between study days 28 and 30. Flea infestations on pets were assessed using visual area thumb counts and premises flea infestations were assessed using intermittent-light flea traps on days 0, 7, 14, 21, and once between study days 28–30, 40–45, and 54–60.

**Results:**

Within 7 days of administration of afoxolaner chewable tablets, flea counts on dogs were reduced by 99.3 %. By one month post-treatment, total flea counts on dogs were reduced by 99.9 %, with 97.3 % (36/37) of the dogs being flea free. Following the second dosing on study day 28–30, total on-dog flea burden was reduced by 100 % on days 40–45 and 54–60. On day 0, the traps collected a geometric mean of 18.2 fleas. Subsequent reductions in emerging flea populations were 97.7 and 100 % by days 28–30 and 54–60, respectively. There were 515 total fleas (*Ctenocephalides felis felis*) collected in the intermittent light flea traps on day 0, and 40.4 % of those fleas displayed visual evidence of having fed. Seven days after initial treatment, only 13.1 % of the fleas contained blood and by day 14 only 4.9 % of the fleas collected in traps displayed evidence of having fed. On day 0, prior to treatment, 60 % of the unfed fleas collected in intermittent-light flea traps were females, but by days 28–30, unfed males accounted for 78 % of the population.

**Conclusions:**

This in-home investigation conducted during the summer of 2014 in subtropical Tampa, FL demonstrated that afoxolaner chewables rapidly and effectively eliminated flea populations in infested dogs and homes.

## Background

Several studies conducted in Tampa, FL (USA) over the past 15 years have demonstrated that dinotefuran-pyriproxyfen, fipronil (±, (s)-methoprene), imidacloprid, indoxacarb, lufenuron (+pyrethrin spray or + nitenpyram tablets) and selamectin were effective in controlling fleas on naturally infested dogs and cats and in private residences within 60 to 90 days, without the need for treatment of premises [1–7]. Premises and on-animal flea infestations are ultimately being controlled in these homes because these products prevent flea reproduction, either killing most newly acquired fleas prior to initiation of egg laying and/or rendering the vast majority of deposited eggs non-viable [5–7]. Field studies such as these and other investigations have demonstrated that flea control likely succeeds or fails based upon a product’s or combination of products’ ability to effectively limit flea reproduction [5–7].

Recently, a new orally administered afoxolaner chewable product was introduced into the flea and tick control market [8–11]. Afoxolaner is a member of the isoxazoline class of compounds and is labeled for the treatment and prevention of flea infestations and treatment and control of ticks on dogs. When afoxolaner was administered to dogs as a single chewable at the minimal effective dose of 2.5 mg/kg it provided 99.9–100 % control of fleas for up to 36 days [8]. Egg production was also almost completely inhibited in that study. The combination of excellent month-long residual adulticide activity and inhibition of egg production would indicate that afoxolaner chewables should provide excellent control of natural flea infestations. The objective of this study was to evaluate the performance of afoxolaner chewables in eliminating natural flea infestations on dogs in Tampa, FL USA.

The effectiveness of the afoxolaner treatment was assessed using both premises and on-animal flea population estimating techniques. Because investigators administered products to all dogs, this study eliminated any potential owner compliance issues.

## Methods

### Home and pet study inclusion criteria

Through referrals from the Sunshine Animal Hospital, Tampa, FL and advertisements on CRAIGSLIST®, 26 private residences were selected for inclusion in the study from May 19–May 29, 2014.

Homes and dogs were selected based on the following criteria: 1) a minimum of five fleas observed in area flea counts on at least one dog at the residence; 2) a minimum of five fleas collected in a 16–24 h period in two intermittent light flea traps; 3) one to five healthy, non-fractious dogs residing at the residence (because no isoxazoline approved for cats was available at the time of this study, households with cats were excluded); 4) qualifying dogs spent ≥ 12 h/day in the indoor premises; 5) homeowner’s willing to participate in the study for at least 2 months; 6) owners agreeing not use any other topical or premises flea control products during the study; 7) owners agreeing not to bring any other dogs into the household for the duration of the study; 8) no dog in the household was pregnant or nursing; 9) qualifying dogs ≥ 8 weeks of age and ≥ 4 lbs; 10) completion of a questionnaire concerning pet habits, visiting pets, previous flea treatments and personal observations around their residence concerning wildlife and feral cats; and 11) no residual flea product was applied in the previous 30 days.

Animals were client-owned dogs and were handled in compliance with Merial Institutional Animal Care and Use Committee and Kansas State IACUC approval (#3413). Throughout the trial, dogs were housed in their normal environment. There were no restrictions other than those normally placed on the pet by the pet-owner.

### Treatment groups

Dogs were treated orally with an afoxolaner chewable (NexGard® Chewables; Merial) according to the label dosing recommendations. Dogs were weighed on Days −1 or 0 and once between days 28–30 to ensure proper dosing. All dogs in each enrolled household were treated on day 0 and then once between days 28–30. All treatments were administered by study investigators. No other topical or premises flea treatments were used during the study. There were no restrictions on the animals with regard to exposure to rain, swimming or movement outdoors. However, while pet activity was not restricted, it was recorded. It should be noted that in some homes with multiple dogs, not all dogs qualified for inclusion in the study (fewer than 5 fleas, resided outdoors, inability to examine, etc.). However, all qualifying and non-qualifying dogs within each enrolled household were still administered a weight appropriate afoxolaner chewable.

This study was conducted without a placebo control group. While the use of a non-treated group might have provided a better evaluation of the performance of the treatment regimen, it is the opinion of these authors that the massive flea infestations commonly encountered in Tampa, FL preclude the use of a non-treated group. Withholding treatment would be detrimental to the health and welfare of the pets and potentially to humans in a household.

### Flea population assessment

The numbers of adult fleas present in the indoor premises were assessed using intermittent light traps [1–7, 12, 13]. One trap was placed in each of two rooms for 16 to 24-h. Rooms were selected based on where the pet(s) spent most of the time or where owners had observed fleas. Once rooms were selected, the traps were returned to the same rooms in the same location at every counting period. Fleas collected on the adhesive pads of the traps were enumerated and identified by microscopic observation as to characteristics (e.g.: fed, unfed, gravid) and as to species. In addition, the sex of fleas collected on light traps for each counting period, for each household, was determined.

The flea population on each pet was assessed using a visual area thumb count methodology [1–7, 14]. Area counts were performed at five locations on each animal; dorsal midline, tail head, left lateral, right lateral, and inguinal region. Area counts were limited to one minute per location and conducted by parting the hair against the lay using both hands until the area was covered. Maximum number of fleas per zone is capped at 50; therefore the maximum total area flea counts for a pet is 250. Pet and premises flea counts were conducted ± 1 day on days 0, 7, 14, 21, then once between days 28–30, 40–45, and 54–60.

### Data analysis

Environmental Control Assessments: Percentage of control achieved by the flea product was calculated using Geometric Means (GM) and the following formula:

$$ \frac{\left(\mathbf{Day}\ \mathbf{z}\ \mathbf{G}\mathbf{M}\ \mathbf{Flea}\ \mathbf{trap}\ \mathbf{Counts}\ \mathbf{\hbox{-}}\ \mathbf{Day}\ \mathbf{y}\ \mathbf{G}\mathbf{M}\ \mathbf{Flea}\ \mathbf{trap}\ \mathbf{Counts}\right)}{\mathbf{Day}\ \mathbf{z}\ \mathbf{G}\mathbf{M}\ \mathbf{Flea}\ \mathbf{trap}\ \mathbf{Counts}\ \mathbf{x}\ \mathbf{100} = \%\ \mathbf{control}} $$

Where z = Day 0

Where y = Days 7, 14, 21, 28–30, 40–45 or 54–60

To determine these geometric means, counts were transformed to the natural logarithm of (count + 1) for calculation.

- **Sex-Ratio & blood fed assessments of fleas collected in intermittent light flea traps:** Fleas collected in traps were counted by sex throughout the study, noting the arithmetic percentages of unfed male to unfed female fleas. Additionally, an assessment was conducted of blood fed and unfed fleas found in the traps. Adhesive sheets were placed under a dissecting microscope and captured fleas were examined for visual evidence of having fed. Determination that a flea had fed (blood consumed) was made by either observing blood in the midgut of the flea or more commonly seeing a droplet or droplets of blood immediately posterior to the flea on the sticky adhesive of the flea trap [5].
- **On-Animal Flea Counts:** On-animal flea count estimates of live adult fleas were transformed to the natural logarithm of (count + 1) for calculation of geometric means at each time point. Percent reduction from the control (Day 0) mean were calculated using the formula [(C-T)/C] × 100, where C = geometric mean for the control count (Day 0) and T = geometric mean for the treated group for each subsequent assessment.

## Results

Twenty-six homes were originally enrolled in the study, but only 23 homes were retained in the study for at least one month. Data from the three households that were not maintained in the study for at least 28–30 days were not included. Two homes were dropped because the owners moved and another household was removed from the study because the owners adopted a cat. Thus, data from twenty-three households were analysed in these assessments.

In the 23 homes that completed the study, there were 37 qualifying dogs (avg. 17.8 kg; range 2.0–47.3 kg) enrolled. On day 0, the dogs received a mean oral dose of 4.09 mg/kg (range 2.59–6.21 mg/kg) afoxolaner. There were an additional 11 dogs in these homes that did not qualify for the study because they: had an insufficient numbers of fleas (<5) on day 0, resided permanently outdoors, or could not be safely handled by flea team members to conduct flea counts. Therefore, there were a total of 48 dogs in the 23 homes that were treated with the afoxolaner chewables. In one home the owners reported they had a visitor dog 7 days into the study. That dog was administered weight appropriate dose of afoxolaner chewable the same day.

Of those 37 officially enrolled dogs, four did not complete the study. Two dogs from the same household were unavailable beyond days 28–30, because the owners moved during the second month of the study. One dog in one household died from canine parvovirus prior to the day 40–45 count. Another dog (14 year old) in another household was reported by the owner as having died from “old age” the week prior to the last visit and was not available for the last flea count. Owners did not report the dog’s death to the K-State Flea Team until the team arrived at the home for the final flea count, by that time the dog had been disposed of by the owners and, therefore, it was impossible to verify the cause of death.

In one household, 5 days after the administration of afoxolaner chewable, the owner reported that one dog was vomiting and had diarrhea. The following day the owner reported that the dog was doing fine. Then again, one week after the second treatment the owner reported that the dog was vomiting and had diarrhea. Again, this resolved within a day. It is unknown if the afoxolaner chewable was related to vomiting and diarrhea episodes 5 to 7 days after its administration. No other dogs experienced any adverse events during the study.

On day 0, the 37 dogs treated with afoxolaner chewables had a geometric mean of 25.1 (range 6–185) fleas observed in area counts (Table 1). Within 7 days of administration of afoxolaner, flea counts were reduced by 99.3 % (Table 1). By 28–30 days post-treatment, the total flea counts were reduced by 99.9 %. In addition, 97.3 % (36/37) of the dogs treated with afoxolaner were flea free within one month. Following re-administration at one month, total on-dog flea burden was reduced by 100 % on days 40–45 and 54–60 (Table 1).

**Table 1:** Geometric mean and percent control of on-animal flea counts in naturally infested homes when dogs were treated with afoxolaner

Treatment group	# dogs day 0		Days post-treatment						
			Day 0	Day 7	Day 14	Day 21	Days 28–30	Days 40–45	Days 54–60
Afoxolaner^1^	37	Geomean^2^	25.12	0.18	0.02	0.06	0.02	0.00	0.00
		(Std dev)	(37.46)	(0.79)	(0.17)	(0.68)	(0.17)	(0.00)	(0.00)
		(Range)	(6–185)	(0–4)	(0–1)	(0–4)	(0–1)	(0–0)	(0–0)
		% control^3^		99.28	99.92	99.74	99.92	100.00	100.00
		% (#) pets with no fleas	0.0 (0/37)	81.1 (30/37)	97.3 (36/37)	94.6 (35/37)	97.3 (36/37)	100 (34/34)	100 (33/33)

^1^Dogs were orally administered an afoxolaner chewable (NexGard®; Chewables; Merial, Inc.) on day 0 and once between days 28–30

^2^Geometric mean numbers of fleas in visual area thumb counts on dogs

^3^{(Day 0 geometric mean animal area flea counts-day x geometric mean animal area flea counts)/day 0 geometric mean animal area flea counts)} × 100

During the entire 2 month study, 739 fleas were trapped in the 23 residences using intermittent-light traps and all were identified as *Ctenocephalides felis felis*, the cat flea. On day 0, the traps collected a geometric mean of 18.2 (range 5–86) fleas (Table 2). Reductions in emerging flea populations were 97.7 and 100 % by days 28–30 and 54–60, respectively (Table 2).

**Table 2:** Geometric mean and percent control of fleas recovered in premises flea traps in naturally infested homes when dogs were treated with afoxolaner

Treatment group	# homes completing study		Days post-Treatment^1^						
			Day 0	Day 7	Day 14	Day 21	Days 28–30	Days 40–45	Days 54–60
Afoxolaner^1^	23	Geomean^2^	18.19	1.69	0.72	1.09	0.43	0.09	0.00
		(Std dev)	(18.01)	(6.26)	(4.65)	(8.87)	(0.89)	(1.28)	(0.00)
		(Range)	(5–86)	(0–26)	(0–17)	(0–43)	(0–3)	(0–6)	(0–0)
		% control^3^		90.73	96.04	93.99	97.66	99.49	100.00

^1^Dogs were orally administered an afoxolaner chewable (NexGard®; Chewables; Merial, Inc.) on day 0 and once between days 28–30

^2^Geometric mean numbers of fleas recovered in two intermittent light flea traps placed in homes

^3^(Day 0 geometric mean trap flea counts-day x geometric mean trap flea counts)/day 0 geometric mean trap flea counts)} × 100

Of particular interest was that 40.4 % of the 515 fleas collected in the intermittent light flea traps on day 0, i.e. prior to treatment, displayed visual evidence of having fed. However, none of the fleas collected throughout the entire study appeared to have undergone prolonged feeding as evidenced by the lack of abdominal enlargement and none of the females were large enough to have reached reproductive capacity.

Following treatment there was not only a reduction in numbers of fleas, but also the percentage of fleas containing blood collected on the traps. Seven days after treatment only 13.1 % of the fleas contained blood and by day 14 only 4.9 % displayed evidence of having fed.

When all homes were averaged together there was a shift over time in the ratio of unfed female to unfed male fleas collected in premises flea traps. On day 0, prior to treatment, 40 % of the unfed fleas collected in intermittent-light flea traps were males. Then on days 7, 14, 21, and 28–30, males accounted for 61, 26, 72 and 78 %, respectively. The percentage of male fleas on days 40–45 was 33 %, but that percentage represents only 2 of 6 fleas from a single home.

The client interviews conducted before homes were entered into the study clearly showed that reservoir hosts for *C. felis* were commonly observed by pet owners. A majority of pet owners said they had seen opossums (65.2 %; 15/23), raccoons (65.2 %; 15/23) and/or feral cats (91.3 %; 21/23) in their yards.

## Discussion

Previous evaluations of the effectiveness of topical and systemic flea products to control natural flea infestations in homes and on pets in Tampa, FL have demonstrated that reductions in total pet flea burdens are almost always less than 100 % following 60 to 90 days of treatment [1–7]. However, in this study afoxolaner chewables achieved 100 % reduction in on-animal flea populations within 40–45 days after two regular monthly administrations. The area count technique used in this and previous in-home investigations has been shown to detect an average of 23.5 % of the total pet flea burden [14]. Therefore, average pretreatment total body flea burdens on dogs were estimated to be 107 fleas based on the geometric mean area count of 25.1.

Within 14 days of the first treatment, a remarkable 97.3 % (36/37) of the dogs were free of fleas. While infestation pressure can vary from year to year it is interesting to note that in two previous investigations in Tampa using this same study design, percent of flea free dogs at similar time frames post-treatment were much lower. At 14 days post-treatment indoxacarb, dinotefuran/pyriproxyfen/permethrin and fipronil (s)-methoprene topical spot-ons had only achieved 71.4 % [6], 20 % [7] and 22.2 % [7] flea free status on treated pets, respectively [6, 7].

It has been previously documented that actively reproducing *Ctenocephalides felis felis* remain resident upon their host and rarely leave unless forced off by insecticide application or host grooming [15]. This permanent association of reproducing fleas with their host is the key factor that allows topical and systemic flea control products to achieve success in eliminating an existing in-home infestation [5–7, 16]. As immature flea life stages continue to develop in the premises, the biomass is ultimately exhausted because effective residual flea products prevent new viable eggs from being deposited [5–7, 16]. While the above is certainly true, recent investigations have brought more refinement to our understanding of this parasite-host interplay. A previous in-home field investigation determined that prior to on-animal treatment 34.4 % of fleas collected in intermittent-light flea traps contained small quantities of blood [5]. Similarly, in this current study 40.4 % of the fleas collected in the intermittent-light flea traps on day 0 displayed visible evidence of having fed. Several factors have been postulated to account for the presence of blood in fleas collected in premises traps such as pet grooming activity, hyper-excitation of fleas due to application of an insecticide, or some of the fleas may have fed upon humans in the household. Another possibility is that before establishing themselves as permanent ectoparasites, there may be a period of time when a percentage of fleas display inter-host movement. This movement between hosts was previously described by Rust (1994) [17], and more recently, another study further demonstrated this inter-host movement of newly acquired non-reproducing *C felis* [18]. In that study, cats were infested with *C. felis* and then 15 min later co-mingled with non-infested cats [18]. Twenty-four hours later it was determined that 20 % of the fleas transferred from the infested to the previously non-infested cats [18]. However, when cats were infested with fleas and held for 48 h before co-mingling with non-infested cats, only 3.7 % of the fleas transferred. In a previous field study only 9 of 771 (1.17 %) fleas collected in the intermittent-light flea traps were considered engorged or to have fed long enough to have potentially reached reproductive status [5] and in the current study none of 515 (0.00 %) fleas collected in the traps displayed evidence of prolonged feeding (engorged) or of having achieved reproductive status. These data indicate that prior to *C. felis* achieving reproductive status (24–48 h), a percentage of the fleas may feed for short durations of time and move on and off the host and that inter-host movement is occurring. Additionally, these data also further support that once reproduction is initiated, movement of fleas on and off the host and inter-host movement is quite limited.

In the context of the current study, it is important to note that there was a reduction in percentage of fed fleas recovered in the flea traps following treatment from 40.4 % prior to treatment, down to 4.9 % on day 14. This reduction in percent of fed fleas recovered in traps following treatment may indicate that most newly emerged fleas jumping on and then feeding on treated dogs were either dying before jumping back off the dogs or were too moribund to jump towards the intermittent light traps. These data, coupled with the high on-animal flea efficacy and percent flea free dogs, demonstrates that afoxolaner chewables provide rapid residual flea kill under field conditions.

Another potential indicator of rapid residual speed of kill of afoxolaner under field conditions can be observed in the single red-line home observed in this study. A “red-line” home is a household in which there is a 20 % or greater increase in emergence flea trap counts, over day 0 trap counts, within one month after treatment [5, 16]. A red-line home indicates that at the time of treatment the flea population is either in a rapid growth phase or development and emergence has been delayed by environmental conditions (i.e.: a period of cool ambient temperatures) [5]. In the one red-line home observed in this study, the premises intermittent-light flea traps counts went from 15 on day 0 to 16 fleas, 17 fleas and then 43 fleas on days 7, 14 and 21, respectively. However, the flea counts on the dog in that household started high at 70 on day 0, but were 0, 0 and 4 on days 7, 14 and 21, respectively, falling to 0 again for the remaining assessments. These data demonstrate that even when there was an escalating emerging flea population, flea numbers on the treated dog did not escalate accordingly. A different measure of product performance entails the evaluation of gender structure of newly emerged (unfed) fleas collected in intermittent-light traps in these homes [5]. Unfed fleas are used in this analysis so that the gender of only the newly emerging fleas is being assessed. While most insect species exhibit protrandry (males tend to emerge before females), *C. felis* belong to a much smaller group that exhibits protogny (females tend to develop before males) [19]. The first fleas to emerge from a cohort of eggs are females, followed by both males and females and then lastly almost exclusively males. It has been previously demonstrated that if flea reproduction is inhibited by insecticidal and/or insect growth regulator treatments administered to a pet, then a sex shift in premises flea population takes place overtime from a female dominated population towards a more male dominated population [5]. In this current study, only 40 % of the unfed fleas collected on day 0 were male, whereas by 28–30 days following treatment 78 % of the unfed fleas collected were male. This was a clear and rapid sex shift indicative of a dramatic reduction, if not complete cessation, of flea reproduction.

A challenge for many pet owners is the flea reinfestation pressure from flea infested urban wildlife as well as dogs and cats. In North America, feral cats and urban wildlife such as opossums and raccoons can be infested with *C. felis*, which can deposit eggs and contaminate protected outdoor premises locations [20]. Given the potential for reinfestation, it should be noted that 91.3 % of dog owners in this study had seen feral cats and 65.2 % also reported observing either opossums or raccoons in their yards. Given that such a large percentage of owners’ yards are frequented by potentially flea infested animals, it is reasonable to assume that these dogs are under frequent infestation pressure.

It has been documented that opossums and feral cats living in urban areas can be infested with *C. felis* infected with *Rickettsia felis* [21–23]*.* It has been established that *Bartonella henselae* is transmitted among cats by *C. felis* and that feral cats may be infected with *Bartonella spp*. [23]. Recently it was demonstrated that raccoons and feral cats in the state of Georgia (USA) had similar rates of *B. henselae* infection as demonstrated by PCR of blood samples [24]. The presence of raccoons, opossums and feral cats in these pet owners’ yards is also important as it relates to the potential transmission of pathogenic *Rickettsia felis* and *Bartonella spp.* to dogs, cats and humans [21–24]. Therefore, a recommendation of continual flea control, in such situations seems prudent not only to prevent re-infestations but also to possibly interdict disease transmission.

## Conclusions

This in-home investigation conducted during the summer of 2014 in subtropical Tampa, FL demonstrated that afoxolaner chewables rapidly and effectively eliminated heavy flea burdens from dogs and in flea-infested homes. On dog flea burdens were near zero within one week, and completely eliminated fleas from all dogs assessed in the study within 6 weeks. The observed sex shift of emerging fleas from predominately female to male and the rapid elimination of emerging fleas in these homes would indicate that afoxolaner greatly reduced, if not completely eliminated, viable flea reproduction.

®NexGard is a registered trademark of Merial.
